# What is the optimal range of fasting stress hyperglycemia ratio for all-cause mortality in American adults: An observational study

**DOI:** 10.1097/MD.0000000000040288

**Published:** 2024-10-25

**Authors:** Jian Li, Chao Yu, Xiaolu Hu

**Affiliations:** a Nanchang 334 Hospital, Department of Critical Care Medicine, Nanchang, Jiangxi, China; b Department of Cardiovascular Medicine, The Second Affiliated Hospital of Nanchang University, Nanchang, Jiangxi, China; c Laboratory Department, Jiangxi Provincial Children’s Hospital, Nanchang, Jiangxi, China.

**Keywords:** all-cause mortality, fasting stress hyperglycemia ratio, NHANES, U-shaped

## Abstract

To date, no studies have been conducted to assess the impact of fasting stress hyperglycemia ratio (SHR) on all-cause mortality. Therefore, the objective of our study is to investigate the association between SHR and all-cause mortality in a population of American adults. The study population was derived from NHANES data spanning from 2005 to 2018. The exposure variable SHR was derived from fasting blood glucose (FBG) and glycosylated hemoglobin (HbA1c%), and the specific calculation formula was as follows: (FBG (mmol/L))/(1.59 × HbA1c (%) − 2.59). The outcome variable was all-cause mortality. A total of 18,457 participants were enrolled in this prospective cohort study. Following a median follow-up period of 90 months, all-cause mortality was observed in 10.32% of the subjects. Cox proportional hazards regression model indicates that there is no significant difference between SHR and all-cause mortality in the fully adjusted model, whether analyzed as a continuous or categorical variable (*P* for trend > 0.05). Through the 2-piecewise Cox proportional hazards regression model, we have determined that the inflection point of SHR in relation to all-cause mortality is 0.88. It has also been observed that when the value of SHR is on the left side of the inflection point (SHR ≤ 0.88), there is a significant 77% (HR: 0.23; 95% CI: 0.10–0.50) reduction in all-cause mortality for each additional unit increase in SHR. Conversely, when the value of SHR exceeds 0.88, there is a substantial 2.40-fold (HR: 2.40; 95% CI: 1.61–3.58) increase in the risk of all-cause mortality (*P* for log likelihood ratio test < .001). The subgroup analysis results demonstrate that sex has the potential to modify the association between SHR and all-cause mortality within the population exhibiting SHR ≤ 0.88. The relationship between SHR and all-cause mortality follows a U-shaped pattern, where in the lowest risk of death for the average American adult is observed at an SHR value of 0.88. Furthermore, in men with SHR ≤ 0.88, there is a significant inverse relationship between the increase in SHR and the risk of all-cause mortality.

**Key Points**

This study is the first to explore the relationship between fasting stress hyperglycemia ratio and all-cause mortalityin a large sample size of the American population.

The relationship between SHR and all-cause mortality follows a U-shaped pattern, where in the lowest risk of death for the average American adult is observed at an SHR value of 0.88.

Furthermore, in men with SHR ≤ 0.88, there is a significant inverse relationship between the increase in SHR and the risk of all-cause mortality.

## 1. Introduction

Diabetes ranks as the 7th leading cause of mortality in the United States, and it is projected by the International Diabetes Federation that there will be a 50% increase in global diabetes prevalence from 366 million individuals in 2011 to an estimated number by 2030.^[1]^ The primary clinical features of diabetes include elevated blood glucose levels and impaired insulin sensitivity.^[2]^ Some critically ill patients with relatively high blood sugar levels, but who do not meet the diagnostic criteria for absolute hyperglycemia of diabetes, are also at an increased risk of mortality.^[3–5]^

The elevation of blood glucose levels in these patients is referred to as stress hyperglycemia, and it is typically assessed using the stress hyperglycemia ratio (SHR).^[6–8]^ The calculation of SHR involves the admission blood glucose and the estimated mean blood glucose, as determined by glycosylated hemoglobin levels.^[6–8]^ Although the specific calculation formula for SHR may vary, it has been shown to significantly increase the risk of mortality in patients with acute myocardial infarction, stroke, and severe illness.^[9–14]^ The admission blood glucose level is influenced by whether or not the patient has eaten, which can result in false positive indications of elevated blood sugar levels. Therefore, it is suggested to substitute the admission blood glucose value with the fasting blood glucose value.^[9,10]^ This would enable extending the applicability of this index to a broader research population. Previous reports suggest that stress hyperglycemia may induce inflammation and oxidative stress, exacerbate endothelial dysfunction, and precipitate a prethrombotic state.^[9]^ These are all known pathophysiological precursors to all-cause mortality. Therefore, examining the relationship between SHR and all-cause mortality, particularly in the context of diabetes exclusivity, is an essential endeavor.

Therefore, we conducted a large-scale cohort study using adult data from the National Health and Nutrition Examination Survey (NHANES) to test our research hypothesis that SHR is significantly associated with all-cause mortality, to assess whether this relationship is linear or nonlinear, and to examine potential effect modifiers. This cohort study aimed to evaluate the predictive ability of SHR for all-cause mortality in the general population and fill existing research gaps.

## 2. Methods

### 2.1. Study design and population

The population of this study comes from NHANES research conducted in the United States. NHANES is a comprehensive cross-sectional survey that aims to assess the health and nutritional status of Americans. NHANES employs a complex, stratified, and multi-stage probability sampling design to ensure representative results. The survey is conducted every 2 years with approximately 5000 American participants selected from various geographic locations across the country. The specific research design used in NHANES has been published elsewhere to provide transparency and allow other researchers to replicate or build upon the findings.^[15,16]^ This ensures that the data collected through this rigorous methodology can be utilized by scientists, policymakers, healthcare professionals, and others interested in understanding public health trends in the United States. Additionally, NHANES’ research plan received approval from the Ethics Committee of National Center for Health Statistics (NCHS), a subsidiary unit of the Centers for Disease Control and Prevention (CDC), and written informed consent was obtained from all participants.

In this analysis, we included all adults aged 18 years or older who participated in the NHANES continuous investigation cycle from 2005 to 2018. A total of 18,457 patients with complete fasting blood glucose (FBG), glycosylated hemoglobin (HbA1c) and endpoint events were included.

### 2.2. Definition of the SHR and outcome ascertainment

The specific formula for SHR can be derived from FBG and HbA1c, as follows: SHR = [(FBG (mmol/L))/(1.59 × HbA1c (%) − 2.59)].^[6]^ The laboratory of Fairview-University Medical Center University Campus conducted tests on FBG and HbA1c. The coefficient of variation for HbA1c falls within the range of 0.7% to 1.2%, thus adhering to the quality control standard of the experiment. For additional information on laboratory testing, please refer to the citedwebsite: https://wwwn.cdc.gov/nchs/data/nhanes/2011-2012/labmethods/ghb_g_met_tosoh_g7.pdf. The study endpoint was all-cause mortality, which was assessed by linking NHANES data with the National Death Index (NDI), a centralized database of death records collected from state vital statistics offices in the United States. Mortality status was primarily determined through probabilistic matching with NDI records. The follow-up period for participants was calculated from the baseline inspection date (2005–2018) until either the registered death date or the end of the study (December 31, 2019), whichever occurred first.

### 2.3. Assessment of covariates

This study screened covariates based on the following criteria: demographic data; variables previously identified as influencing SHR and all-cause death; consistent with STROBE guidelines, covariates were included if they resulted in a change of more than 10% in the basic model^[17]^; other variables were selected based on clinical practice.

Our covariates comprise age, sex, race, body mass index (BMI), systolic blood pressure (SBP) and diastolic blood pressure (DBP), smoking status, poverty income ratio, biochemical indicator, medical history and medication use. The biochemical indices encompass FBG, HbA1c, total cholesterol (TC), triglycerides (TG), high-density lipoprotein (HDL), low-density lipoprotein (LDL), serum uric acid (SUA), total protein and estimated glomerular filtration rate (eGFR) calculated using the Chronic Kidney Disease Epidemiology Collaboration (CKD-EPI) formula.^[18]^ The definition of diabetes mellitus (DM) in this study encompassed self-reported physician diagnosis, FBG levels equal to or exceeding 7 mmol/L, or current usage of medication specifically designed to lower blood glucose levels. The definition of hypertension included self-reported physician diagnosis, a SBP ≥ 140 mm Hg and/or DBP ≥ 90 mm Hg, or the use of antihypertensive medication.^[19]^ Cardiovascular diseases (CVD) in our study were defined as any reported diagnosis of coronary heart disease, angina pectoris, myocardial infarction, chronic heart failure, and stroke.^[20]^

### 2.4. Statistical analysis

We adhered to the statistical analysis guidelines recommended by the CDC for our research data.^[21]^ In the descriptive analysis of baseline data, we stratified SHR into 3 groups. Continuous variables were presented as mean ± standard deviation (SD), while categorical variables were expressed as numbers and percentages. One-way ANOVA was used to compare continuous variables among the 3 groups of SHR, while Chi-square test was employed for categorical variables. We constructed 2 Cox proportional hazards regression model to investigate the association between SHR levels and mortality: Model 1 was adjusted for age, sex, race, poverty income ratio, BMI, SBP, DBP, current smoking, CVD, DM, hypertension, antihypertensive drugs, lipoprotein-lowering drugs, hypoglycemic drugs, total protein; Model 2 was adjusted for age, sex, race, poverty income ratio, BMI, SBP, DBP, TG, TC, HDL, LDL, SUA, total protein, eGFR, current smoking, CVD, DM, hypertension, antihypertensive drugs, lipoprotein-lowering drugs, hypoglycemic drugs. Furthermore, restricted cubic splines with 4 knots were used to flexibly model and visualize the dose-response relationship between SHR levels and mortality. We tested for potential non-linearity by using a likelihood ratio test comparing the model with only a linear term against the model with linear and cubic spline terms.^[22–24]^ In the presence of a non-linear relationship, an inflection point between SHR and all-cause death is determined using a recursive algorithm. Two-piecewise Cox proportional hazard models are then employed to analyze the relationship between SHR and all-cause death risk on either side of the inflection point. Furthermore, we conducted subgroup analyses and interaction tests. Specifically, within populations with SHR ≤ 0.88 and SHR > 0.88, we examined potential effect modifiers between SHR and all-cause mortality in the following subgroups: sex (men vs women), age (<65 vs ≥65 years), BMI (<25 vs ≥25 kg/m^2^), smoking status (never vs former vs current), hypertension (no vs yes), and DM (no vs yes). We conducted a sensitivity analysis, in which the left-skewed FBG was transformed using a log10 function. We then assessed the impact of LgFBG on all-cause mortality using a 2-piecewise Cox proportional hazard regression analysis. The statistical analysis of this study was conducted using R software and Empower Stats (http://www.empowerstats.com, X&Y Solutions, Inc., Boston). A bilateral *P* value <.05 was considered statistically significant.

## 3. Results

### 3.1. Baseline characteristics

A total of 18,457 participants were enrolled in this prospective cohort study, with males accounting for 48.59% and the average age (SD) being 48.20 (SD: 18.76) years. Following a median follow-up period of 90 months, all-cause mortality was observed in 10.32% of the subjects. Table 1 presents the baseline characteristics of the study population stratified by third-class grouping based on SHR values. The participants had a mean (SD) SHR of 0.93 (0.14). No significant difference in serum total protein levels was observed among the 3 groups, while statistically significant differences were found in other indices. Patients with the highest SHR (≥0.96) exhibit several characteristics, including being male, younger, having fewer current smokers and a higher incidence of complications such as DM, CVD and hypertension. In addition, compared to the higher group of patients, patients with SHR < 0.87 exhibit elevated BMI, FBG, TC, HDL, and DL levels; decreased SBP, DBP, poverty income ratio, HbA1c%, TG, SUA and eGFR levels; a higher prevalence of antihypertensive and lipid-lowering drug usage; and a lower prevalence of hypoglycemic drug usage. We also observed significant disparities with regard to race. In the group with the highest SHR, non-Hispanic whites were the most prevalent, followed by Mexican Americans, with non-Hispanic Blacks ranking third, and individuals of other races being the least prevalent.

**Table 1:** Baseline characteristics of the study population.

Characteristics	SHR			*P* value
	Tertile 1 (<0.87)	Tertile 2 (0.87–0.96)	Tertile 3 (≥0.96)	
N	6141	6148	6168	
Males, N (%)	2522 (41.07%)	2877 (46.80%)	3570 (57.88%)	<.001
Age (yr)	49.63 ± 18.66	47.25 ± 18.68	47.73 ± 18.86	<.001
BMI (kg/m^2^)	28.73 ± 7.03	28.71 ± 6.84	29.40 ± 7.10	<.001
Race				<.001
Non-Hispanic White, N (%)	2129 (34.67%)	2670 (43.43%)	2890 (46.85%)	
Non-Hispanic Black, N (%)	1891 (30.79%)	1033 (16.80%)	947 (15.35%)	
Mexican American, N (%)	832 (13.55%)	1053 (17.13%)	1114 (18.06%)	
Other Hispanic, N (%)	603 (9.82%)	621 (10.10%)	599 (9.71%)	
Other races, N (%)	686 (11.17%)	771 (12.54%)	618 (10.02%)	
Current smoking, N (%)	1294 (21.80%)	1164 (19.64%)	1137 (19.20%)	<.001
SBP (mm Hg)	122.68 ± 19.29	121.59 ± 18.12	124.05 ± 18.27	<.001
DBP (mm Hg)	68.39 ± 12.15	69.02 ± 11.81	69.78 ± 12.18	<.001
Poverty income ratio	2.38 ± 1.59	2.52 ± 1.64	2.47 ± 1.63	<.001
FBG (mg/dL)	97.06 ± 20.38	104.15 ± 20.83	125.63 ± 51.16	<.001
HbA1c (%)	5.92 ± 1.01	5.62 ± 0.79	5.69 ± 1.39	<.001
TC (mg/dL)	192.08 ± 42.75	192.56 ± 41.98	188.19 ± 41.62	<.001
TG (mg/dL)	115.94 ± 87.03	121.36 ± 107.85	139.24 ± 126.28	<.001
HDL (mg/dL)	56.15 ± 16.40	54.46 ± 15.92	51.75 ± 15.83	<.001
LDL (mg/dL)	113.01 ± 36.39	114.00 ± 35.47	109.75 ± 34.87	<.001
SUA (mg/dL)	5.37 ± 1.47	5.43 ± 1.40	5.61 ± 1.45	<.001
Total protein (mg/dL)	7.15 ± 0.49	7.15 ± 0.47	7.16 ± 0.47	.551
eGFR (mL/min/1.73 m^2^)	94.93 ± 25.94	97.73 ± 23.76	96.65 ± 24.33	<.001
Comorbidities, N (%)				
DM	901 (15.01%)	656 (10.84%)	1676 (27.60%)	<.001
CVD	722 (12.38%)	596 (10.28%)	705 (12.17%)	<.001
Hypertension	2571 (41.87%)	2276 (37.02%)	2661 (43.14%)	<.001
Medication use, N (%)				
Antihypertensive drugs	2009 (32.71%)	1686 (27.42%)	1977 (32.05%)	<.001
Lipoprotein-lowering drugs	1329 (21.64%)	1055 (17.16%)	1246 (20.20%)	<.001
Hypoglycemic drugs	761 (12.39%)	436 (7.09%)	950 (15.40%)	<.001

BMI = body mass index, CVD = cardiovascular diseases, DBP = diastolic blood pressure, DM = diabetes mellitus, eGFR = estimated glomerular filtration rate, FBG = fasting blood glucose, HbA1c = glycosylated hemoglobin, HDL = high-density lipoprotein cholesterol, LDL = low-density lipoprotein cholesterol, SBP = systolic blood pressure, SUA = serum uric acid, TC = total cholesterol, TG = triglyceride.

### 3.2. Association between fasting SHR and all-cause mortality

**Figure 1.**
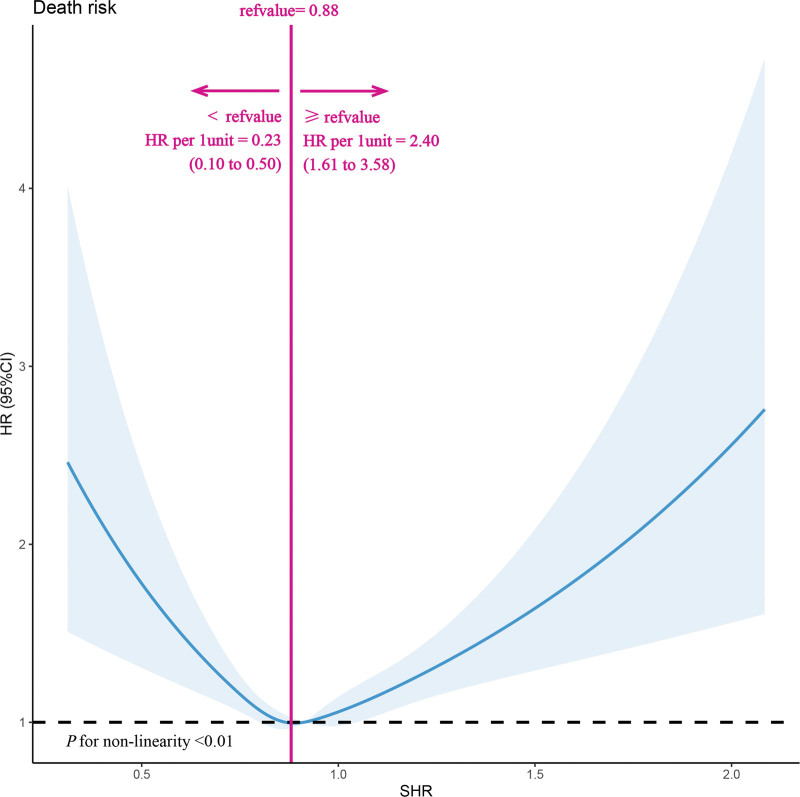
Dose-response relationship betwee SHR and all-cause mortality. A nonlinear association between SHR and all-cause mortality was found. The solid line and dashed line represent the estimated values and their corresponding 95% confidence interval. Adjustment factors included age, sex, race, poverty income ratio, BMI, SBP, DBP, TG, TC, HDL, LDL, SUA, total protein, eGFR, current smoking, CVD, DM, hypertension, antihypertensive drugs, lipoprotein-lowering drugs, hypoglycemic drugs. BMI = body mass index, CVD = cardiovascular diseases, DBP = diastolic blood pressure, DM = diabetes mellitus, eGFR = estimated glomerular filtration rate, HDL = high-density lipoprotein cholesterol, LDL = low-density lipoprotein cholesterol, SBP = systolic blood pressure, SHR = stress hyperglycemia ratio, SUA = serum uric acid, TC = total cholesterol, TG = triglyceride.

As depicted in Figure 1, a U-shaped association is observed between fasting SHR and all-cause mortality, indicating that both elevated and reduced levels of SHR significantly elevate the risk of all-cause mortality. Simultaneously, we employ the Cox proportional hazard regression model to assess the impact of SHR on all-cause mortality and develop 2 models, namely model 1 and model 2. Table 2 indicates that there is no significant difference between SHR and all-cause mortality in the fully adjusted model 2, whether analyzed as a continuous or categorical variable (*P* for trend > .05). The specific data are as follows: In comparison to the T1 group of SHR, the adjusted HR for all-cause mortality in the T2 and T3 groups were 1.00 (95% CI: 0.88–1.14) and 1.06 (95% CI: 0.93–1.20), respectively (model 2). The findings indicate a non-linear association between SHR and all-cause mortality, suggesting that the Cox proportional hazard regression model is not appropriate for capturing their relationship. Two-piecewise Cox proportional hazards regression model was used to calculate the threshold effect of the SHR. If the log likelihood ratio test < 0.05, it means the 2-piecewise Cox proportional hazards regression model is superior to the single-line logistic regression model. Through the aforementioned model, we have determined that the inflection point of SHR in relation to all-cause mortality is 0.88. It has also been observed that when the value of SHR is on the left side of the inflection point (SHR ≤ 0.88), there is a significant 77% (HR: 0.23; 95% CI: 0.10–0.50) reduction in all-cause mortality for each additional unit increase in SHR. Conversely, when the value of SHR exceeds 0.88, there is a substantial 2.40-fold (HR: 2.40; 95% CI: 1.61–3.58) increase in the risk of all-cause mortality (*P* for log likelihood ratio test < .001; Table 3). Additionally, our sensitivity analysis of fasting FBG reveals a U-shaped correlation between FBG and all-cause mortality, similar to that observed with SHR. The cutoff point for all-cause mortality is observed at LgFBG = 2.14 (FBG = 96 mg/dL). This suggests that when LgFBG > 2.14, there is a significant rise in the risk of all-cause mortality commensurate with increasing FBG levels (HR: 7.37; 95% CI: 2.87–18.89; Table S1, Supplemental Digital Content, http://links.lww.com/MD/N818).

**Table 2:** Association between fasting SHR and all cause mortality among US adults.

All cause mortality HR (95% CI), *P* value	SHR				
	Tertile 1 (<0.87)	Tertile 2 (0.87–0.96)	Tertile 3 (≥0.96)	*P* for trend	Per 1 unit increase
Events (%)	661 (10.76%)	542 (8.82%)	701 (11.37%)		1904 (10.32%)
Model 1	Reference	0.98 (0.86, 1.11) 0.709	1.04 (0.92, 1.19) 0.507	.521	1.31 (0.98, 1.76) 0.070
Model 2	Reference	1.00 (0.88, 1.14) 0.979	1.06 (0.93, 1.20) 0.397	.407	1.28 (0.92, 1.78) 0.146

Model 1 was adjusted for age, sex, race, poverty income ratio, BMI, SBP, DBP, current smoking, CVD, DM, hypertension, antihypertensive drugs, lipoprotein-lowering drugs, hypoglycemic drugs, total protein.

Model 2 was adjusted for age, sex, race, poverty income ratio, BMI, SBP, DBP, TG, TC, HDL, LDL, SUA, total protein, eGFR, current smoking, CVD, DM, hypertension, antihypertensive drugs, lipoprotein-lowering drugs, hypoglycemic drugs.

BMI = body mass index, CVD = cardiovascular diseases, DBP = diastolic blood pressure, DM = diabetes mellitus, eGFR = estimated glomerular filtration rate, HDL = high-density lipoprotein cholesterol, HR = hazard ratio, LDL = low-density lipoprotein cholesterol, SBP = systolic blood pressure, SHR = stress hyperglycemia ratio, SUA = serum uric acid, TC = total cholesterol, TG = triglyceride.

**Table 3:** Results of 2-piecewise Cox proportional hazards regression model.

SHR	All cause mortality HR (95% CI)*, *P* value
Inflection point (K)	.88
≤0.88	0.23 (0.10, 0.50) *P* = .0002
>0.88	2.40 (1.61, 3.58) *P* < .0001
*P* for log likelihood ratio test	<.001

Two-piecewise Cox proportional hazards regression model was used to calculate the threshold effect of the SHR. If the log likelihood ratio test > 0.05, it means the 2-piecewise Cox proportional hazards regression model is not superior to the single-line logistic regression model.

BMI = body mass index, CVD = cardiovascular diseases, DBP = diastolic blood pressure, DM = diabetes mellitus, eGFR = estimated glomerular filtration rate, HDL = high-density lipoprotein cholesterol, LDL = low-density lipoprotein cholesterol, SBP = systolic blood pressure, SHR = stress hyperglycemia ratio, SUA = serum uric acid, TC = total cholesterol, TG = triglyceride.

* Adjusted for age, sex, race, poverty income ratio, BMI, SBP, DBP, TG, TC, HDL, LDL, SUA, total protein, eGFR, current smoking, CVD, DM, hypertension, antihypertensive drugs, lipoprotein-lowering drugs, hypoglycemic drugs.

### 3.3. Subgroup analysis

**Figure 2.**
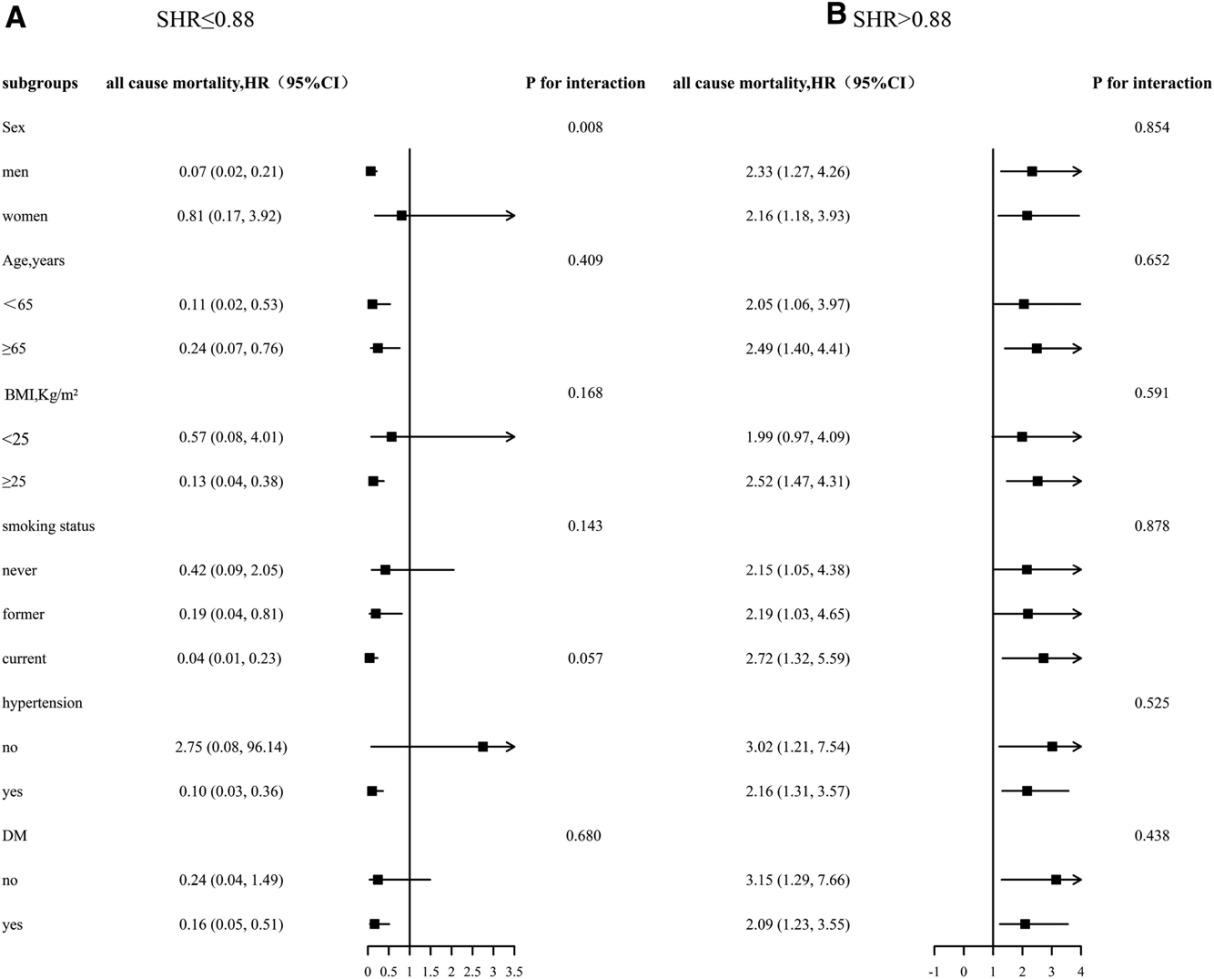
Stratified analysis for the SHR and all-cause mortality in various subgroups divided by 0.88. (A) SHR ≤ 0.88, (B) SHR > 0.88. Each subgroup analysis adjusted, if not stratified, for age, sex, race, poverty income ratio, BMI, SBP, DBP, TG, TC, HDL, LDL, SUA, total protein, eGFR, current smoking, CVD, DM, hypertension, antihypertensive drugs, lipoprotein-lowering drugs, hypoglycemic drugs. BMI = body mass index, CVD = cardiovascular diseases, DBP = diastolic blood pressure, DM = diabetes mellitus, eGFR = estimated glomerular filtration rate, HDL = high-density lipoprotein cholesterol, LDL = low-density lipoprotein cholesterol, SBP = systolic blood pressure, SHR = stress hyperglycemia ratio, SUA = serum uric acid, TC = total cholesterol, TG = triglyceride.

In the stratified analysis, we will categorize SHR into 2 groups based on a cutoff point of 0.88 and examine its impact on all-cause mortality within subgroups defined by sex, age, BMI, smoking status, hypertension, and diabetes (Fig. 2). No significant interaction was observed between SHR and the stratified variables when SHR > 0..88 (Fig. 2B). In the SHR ≤ 0.88 side, we exclusively observed a more pronounced negative correlation between SHR and all-cause mortality in men (*P* for interaction = .008). However, no similar findings were observed in other variable subgroups (Fig. 2A).

## 4. Discussion

In this large-scale observational cohort study, we discovered a significant association between SHR and all-cause mortality in the general population, exhibiting a U-shaped correlation. Furthermore, our analysis revealed that the inflection point of SHR was determined to be 0.88. Notably, deviations from this value on either side were found to amplify the risk of all-cause death. Additionally, the subgroup analysis results demonstrate that sex has the potential to modify the association between SHR and all-cause mortality within the population exhibiting SHR ≤ 0.88.

The application scope of the SHR index has been previously demonstrated to be limited to patients with acute myocardial infarction (AMI),^[9,10,12]^ those in critical illness,^[13,14]^ and for prognosis research of stroke patients.^[11]^ The definition of SHR is also inconsistent. The specific algorithms are as follows: FBG/ HbA1c,^[8]^ admission BG (mmol/L)/(1.59 × HbA1c (%) − 2.59)^[6]^ and the definition of FBG (mmol/L)/(1.59 × HbA1c(%) − 2.59).^[9]^ HbA1c serves as a reliable indicator for assessing blood glucose levels over the preceding 8 to 12 weeks, enabling estimation of the average glucose concentration during this period.^[25,26]^ Furthermore, given that admission blood glucose is influenced by dietary intake, continuous refinement of the SHR formula has expanded its applicability and enhanced the utility of this index. According to the definition of SHR in this study, Fu et al^[10]^ and Cui et al^[9]^ both utilized the China Acute Myocardial Infarction Registration Study to investigate the association between SHR and in-hospital mortality. The study conducted by Fu et al revealed that in a cohort of 5308 AMI patients, the optimal cutoff values of SHR for predicting in-hospital mortality differed between diabetic and non-diabetic patients, with thresholds of 1.06 and 1.26 respectively. Furthermore, irrespective of diabetes status, there was a positive association observed between SHR levels and the occurrence of in hospital death.^[10]^ Cui et al conducted a comparative analysis of the predictive ability of SHR, FBG and HbA1c for hospitalization death in AMI patients with varying blood glucose levels. The findings revealed that SHR significantly increased the risk of hospitalization death among AMI patients with different blood glucose statuses, and its predictive power for hospitalization death was higher than that of FBG in diabetic AMI patients.^[9]^ A study conducted using the critical care database examined the association between SHR and all-cause mortality. This retrospective analysis encompassed 3636 patients admitted to the intensive care unit. Following a 1-year follow-up period, it was observed that SHR was significantly associated with both ICU mortality and 1-year all-cause mortality in critically ill patients, exhibiting an increasing predictive value across various disease scores. Furthermore, our findings revealed a higher risk of all-cause mortality among non-diabetic individuals compared to diabetic patients.^[13]^

In contrast to this study, all aforementioned studies demonstrate a significant positive correlation between SHR and mortality. This may be attributed to the fact that the patients in those studies were in a severe disease state with high baseline levels of SHR, and the follow-up duration was too short to ascertain long-term prognosis of SHR. This study represents the first evaluation of the impact of SHR on all-cause mortality in a general American adult population, expanding the potential application of this indicator. Through retrospective cohort analysis, we identified a U-shaped correlation between SHR and all-cause mortality. The lowest risk of all-cause death was observed at an SHR value of 0.88 (8.82%). As the distance from this cut point increased, so did mortality rates to 10.76% and 11.37%, respectively. Notably, when approaching an inflection point at 0.88, we found that risk decreased by 77% (HR: 0.23, 95% CI: 0.10–050) to the left while increasing by a factor of 2.40 (HR: 2.40, 95% CI: 1.61–3) to the right. Additionally, we observed sex-specific variations in the impact of SHR on all-cause mortality. Specifically, among participants with an SHR less than or equal to 0.88, a negative correlation between SHR and all-cause death was only evident in men. This finding may be attributed to the lower baseline SHR levels observed in men compared to women within our study population. Notably, excessively low SHR levels may indicate hypoglycemia and consequently increase the risk of mortality.^[27,28]^ Therefore, maintaining an optimal blood sugar range within the normal population necessitates identifying a safe range for SHR values. The increase in SHR may potentially contribute to an elevated risk of mortality through the following mechanisms: impairment of insulin secretion by pancreatic β cells, which fails to counteract the hyperglycemic effects induced by anti-regulatory hormones and cytokines, resulting in stress hyperglycemia.^[29,30]^ Furthermore, sympathetic nervous system activation exacerbates insulin resistance by promoting the release of free fatty acids (FFAs) from adipose tissue and stimulating serine/threonine kinases that disrupt insulin signal transduction.^[31,32]^ Importantly, stress-induced hyperglycemia can directly lead to adverse consequences by inducing endothelial dysfunction and oxidative stress.^[3]^

This study is the first time to observe the long-term effect of SHR on all-cause death in American adults, and find out that the lowest tangent point of SHR is 0.88. In clinical practice, the SHR level of adults can be monitored. Those with values exceeding 0.88 could be classified as high-risk. Following this, further examination can be conducted to ascertain whether the individual has concomitant traditional cardiovascular risk factors. This would allow for timely clinical intervention aimed at mitigating the risk of mortality.

## 5. Limitations

The findings of this study possess certain limitations and should be cautiously interpreted. Firstly, it is important to note that this study follows a retrospective observational design. Although we have employed rigorous statistical methodologies to mitigate bias, further investigations are warranted to elucidate the association between SHR and all-cause mortality. Secondly, it is crucial to acknowledge that our research sample solely comprises average American adults, thus caution must be exercised when generalizing these conclusions to other populations under investigation.

## 6. Conclusions

The findings of a retrospective cohort study, conducted on a large research population, demonstrate that both high and low systolic-to-heart-rate ratios (SHR) are associated with an increased risk of mortality among American adults, with an optimal SHR value of 0.88. Furthermore, in men with SHR ≤ 0.88, there is a significant inverse relationship between the increase in SHR and the risk of all-cause mortality.

## Acknowledgments

A special thanks to all of the NHANES participants who freely gave their time to make this and other studies possible.

## Author contributions

**Data curation:** Chao Yu.

**Funding acquisition:** Xiaolu Hu.

**Investigation:** Xiaolu Hu.

**Methodology:** Jian Li.

**Writing – original draft:** Jian Li.

## Supplementary Material


