# Supplementary material for: What is the optimal range of fasting stress hyperglycemia ratio for all-cause mortality in American adults: An observational study
Source: Medicine (Baltimore). 2024 Oct 25;103(43):e40288. doi: 10.1097/MD.0000000000040288 (PMC11521012; doi:10.1097/MD.0000000000040288)
Supplement: Supplementary file 1 [file medi-103-e40288-s001.docx]

Supplementary material

Supplementary Table1 Results of two-piecewise Cox proportional hazards regression model

| LgFBG (mg/dL) | all cause mortality HR (95%CI)^#^, P value |
| --- | --- |
| Inflection point (K) | 2.14 |
| ≤2.14 | 0.31 (0.11, 0.89) P=0.03 |
| >2.14 | 7.37 (2.87, 18.89) P<0.0001 |
| *P* for log likelihood ratio test | <0.001 |

Two-piecewise Cox proportional hazards regression model was used to calculate the threshold effect of the LgFBG. If the log likelihood ratio test >0.05, it means the two-piecewise Cox proportional hazards regression model is not superior to the single-line logistic regression model.

^#^Adjusted for age, sex, race, poverty income ratio, BMI, SBP, DBP, TG, TC, HDL, LDL, SUA, total protein, eGFR, current smoking, CVD, DM, hypertension, antihypertensive drugs, lipoprotein-lowering drugs, hypoglycemic drugs.
